# Leveraging training expertise to build capacity in computational personalised medicine

**DOI:** 10.1093/bioadv/vbag070

**Published:** 2026-04-25

**Authors:** Marta Lloret-Llinares, Daniel Thomas-Lopez, José Carbonell-Caballero, Laurence Calzone, Javier Conejero, Jesse P Harrison, Miroslav Kratochvil, Arnau Montagud, Vincent Noël, Henrik Nortamo, Miguel Ponce-de-León, Pablo Rodríguez-Mier, Marco Ruscone, Dénes Türei, Miguel Vazquez, Alessandra Villa, Nadja Zlender, Brane Leskosek, Mariano Vazquez, The PermedCoE consortium, Alba Jene-Sanz, Thaleia Ntiniakou, Salvador Capella-Gutierrez, Rosa M Badia, Othmane Hayoun, José Estragués, Adam Šmelko, David Vicente, Gaurav Saxena, Marta Garcia-Gasulla, Marc Clascà, Renata Gimenez, Janine Gehrig, Romana Kronjevod, Esther Dorado, Mariola Tarrega, Laura Portell, Asier Gonzalez, Wei Gu, Sarah Peter, Adrian Thorogood, Laurent Heirendt, Attila Gabor, Francisco Javier Nieto, María Alejandra Paz, Jesus Gorronogoitia, Damjana Kastelic, Joaquim Calbó, Rossen Apostolov, Reinhard Schneider, Julio Saez-Rodriguez, Tommy H Nyrönen, Emmanuel Barillot, Alfonso Valencia, Vera Matser, Cath Brooksbank

**Affiliations:** EMBL’s European Bioinformatics Institute, Wellcome Genome Campus, Hinxton, Cambridge, CB10 1SD, United Kingdom; EMBL’s European Bioinformatics Institute, Wellcome Genome Campus, Hinxton, Cambridge, CB10 1SD, United Kingdom; Barcelona Supercomputing Center (BSC), Barcelona, 08034, Spain; Institut Curie, Université PSL, Paris, F-75005, France; Barcelona Supercomputing Center (BSC), Barcelona, 08034, Spain; CSC—IT Center for Science Ltd, Espoo, FI-02101, Finland; Luxembourg Centre for Systems Biomedicine, University of Luxembourg, Belvaux, L-4367, Luxembourg; Barcelona Supercomputing Center (BSC), Barcelona, 08034, Spain; Institute for Integrative Systems Biology (I2SysBio), CSIC-UV, Valencia, 46980, Spain; Institut Curie, Université PSL, Paris, F-75005, France; CSC—IT Center for Science Ltd, Espoo, FI-02101, Finland; Barcelona Supercomputing Center (BSC), Barcelona, 08034, Spain; Heidelberg University, Faculty of Medicine, and Heidelberg University Hospital, Institute for Computational Biomedicine, Heidelberg, 69120, Germany; Barcelona Supercomputing Center (BSC), Barcelona, 08034, Spain; Institut Curie, Université PSL, Paris, F-75005, France; Heidelberg University, Faculty of Medicine, and Heidelberg University Hospital, Institute for Computational Biomedicine, Heidelberg, 69120, Germany; Barcelona Supercomputing Center (BSC), Barcelona, 08034, Spain; KTH, Royal Institute of Technology, SE-100 44, Stockholm, Sweden; University of Ljubljana, Ljubljana, SI-1104, Slovenia; University of Ljubljana, Ljubljana, SI-1104, Slovenia; Barcelona Supercomputing Center (BSC), Barcelona, 08034, Spain; Barcelona Supercomputing Center (BSC), Barcelona, 08034, Spain; ICREA, Barcelona, 08010, Spain; EMBL’s European Bioinformatics Institute, Wellcome Genome Campus, Hinxton, Cambridge, CB10 1SD, United Kingdom; EMBL’s European Bioinformatics Institute, Wellcome Genome Campus, Hinxton, Cambridge, CB10 1SD, United Kingdom

## Abstract

**Summary:**

Rapid development of genomic technologies in recent years enables personalised medicine to become an essential part of healthcare. Advanced computational methods are required to extract relevant insights that can be applied in clinical settings. This presents a challenge for clinicians and biomedical researchers, who need specialised training to adopt these tools. Within the context of PerMedCoE, the first European Centre of Excellence in Personalised Medicine, we developed and delivered a competency-based training programme to support professionals in the life sciences to work with modelling and simulation tools that integrate omics data to identify biological processes relevant to disease. We identified a set of required competencies in the field and built a series of career profiles with specific competence levels in these. The competencies and profiles contributed to define the focus and target audience of the training activities delivered: a combination of self-paced learning resources, webinars and online and face-to-face synchronous courses. The outputs of the programme (competencies, career profiles and training materials) can be used by biomedical professionals for their own career development or to train others. In addition, the approach can be adopted by other fields with rapid technological advancements and a constant need to upskill professionals.

**Availability and implementation:**

The competency framework is reproduced in full in this paper as supplementary material and available on the Competency Hub at https://competency.ebi.ac.uk/framework/permedcoe/2.1.

## 1 Introduction

Personalised medicine has become a cornerstone of modern healthcare, fueled by rapid advancements in genomic technologies, such as single-cell sequencing. These emerging technologies enable highly detailed and individualised analyses of genomic profiles, opening new avenues for targeted therapies and precision treatments. However, the integration of these technologies introduces significant challenges in data processing and interpretation, particularly in generating mechanistic hypotheses from the vast, complex, and often heterogeneous datasets they produce. The multidimensional nature of omics data requires advanced computational methods to extract biologically relevant insights that can be applied in real-world clinical scenarios.

In response to these challenges, PerMedCoE was established as the first European Centre of Excellence in Personalised Medicine, conceived to create a comprehensive roadmap to advance computational frameworks and software tools for the mechanistic interpretation of personal genomic data within clinically relevant timeframes. These tools are designed to integrate and analyse large-scale omics datasets, facilitating the identification of underlying biological processes, molecular pathways, and personalised therapeutic targets. A critical aspect of this effort involves adapting these simulation tools to high-performance computing (HPC) environments, ensuring scalability and computational efficiency to derive clinically actionable models from large-scale datasets. The growing complexity of these tools presents a challenge for biomedical and clinical professionals, and requires specialised training efforts to support their adoption within the broader scientific community.

The development of these computational frameworks and tools requires an interdisciplinary approach, in which professionals from different backgrounds such as biomedical researchers, clinicians, mathematicians, and computational scientists, work together. All of them need to acquire basic understanding of the other disciplines to be able to communicate and collaborate with each other (Helikar *et al.* 2015, Beckmann and Lew 2016, Helikar 2021, UKRI 2022). Leveraging the expertise of the consortium partners, spanning computational science, mathematics, physics, biology and training, PerMedCoE delivered a competency-based training programme so that professionals in the biomedical sciences could build the required knowledge and skills to work on modelling and simulations, including the use of the software developed by the consortium. This programme aimed to be effective and inclusive and, therefore, was guided by recommendations and principles agreed by training experts, such as the bicycle principles (Williams *et al.* 2023), an actionable framework to improve short-format training based on research in education, and the guidance on applying FAIR principles to training materials (Garcia *et al.* 2020).

PerMedCoE defined a list of relevant competencies for professionals working in computational personalised medicine. The creation of this competency framework was aligned with other efforts to establish common standards and a shared language on skills and occupations in research, such as the ones led by the International Society for Computational Biology (ISCB) (Mulder *et al.* 2018, Brooksbank *et al.* 2024), BioExcel (Matser 2016, Llinares *et al.* 2019), and the European Union (https://research-and-innovation.ec.europa.eu/jobs-research/researchcomp-european-competence-framework-researchers_en). A competency-based approach to training engenders focus on the specific abilities required in the field. PerMedCoE complemented this approach with a needs analysis, to prioritise which competencies to focus on and, therefore, to optimise the use of project resources.

The rapid development of technologies demands continuous learning from researchers and healthcare professionals. The competency-based approach presented here can guide professionals in their career development and be adapted by organisations to design training in their context.

## 2 Development of the competency framework

### 2.1 Definition of competencies

The PerMedCoE competency profile was developed by experts from the consortium, taking as a basis the work done in several related initiatives. These included the competency framework for bioinformatics professionals developed by the International Society for Computational Biology to provide a standard for the extensive education and continuous professional development required in this area (Mulder *et al.* 2018, Brooksbank *et al.* 2024) and the competencies defined by the EU-funded initiatives BioExcel (https://competency.ebi.ac.uk/framework/bioexcel/2.0) and CINECA (https://competency.ebi.ac.uk/framework/cineca/1.0). The effort is aligned with the development by the European Union of a competency framework for researchers with the ambition of enabling ‘widespread recognition of the competences and career development of researchers in various stages of their careers’ and with the aim of supporting self-assessment, education and training, and job seeking (European Commission E 2022).

A multidisciplinary working group within the consortium prepared an initial draft tailored to PerMedCoE and its user communities, with a particular focus on biomedical researchers involved in cellular-level simulations across disciplines such as biology, computer science and physics. During the consortium General Assembly meeting in May 2021, a dedicated session was organised with all the partners to gather feedback on the draft. The input received was then incorporated into version 1.0, which contained 19 competencies divided into three domains: Computational personalised medicine, General computing, and Parallel computing.

A competency profile needs to be updated as new developments in the field emerge or as the community uses it and finds ways to improve it. The use of the PerMedCoE competency profile indicated that it would be useful to align it with the competencies from BioExcel, the European Centre of Excellence in Biomolecular research, as both initiatives develop simulation software in the life sciences, which aims at modelling experimental observations with mathematical objects. While PerMedCoE focuses on simulations at the cellular and tissue level, BioExcel focuses on biomolecular simulations. A working group formed by experts from both initiatives refined the competencies in the ‘General computing’ and ‘Parallel computing’ domains so that they could be used by both communities. The main changes to achieve this were to remove references to the specific areas of focus, e.g. biomolecular simulations. This led to version 2.0 of the competency framework for computational personalised medicine developed by PerMedCoE (Table 1), which includes competencies such as ‘Write or adapt scripts and computer programs (software development) to perform simulations in compliance with good programming practice’. Each competency is further detailed with a series of attributes defining the knowledge, skills, and attitudes required to demonstrate competency (Table S1).

**Table 1:** List of competencies and career profiles and their competence levels in the framework for computational personalised medicine.

		PhD student in computational systems biology	PhD student in biomedicine	Postdoctoral fellow in computational systems biology	Postdoctoral fellow in molecular biology	Postdoctoral fellow in molecular dynamics simulations	Biomedical researcher	Senior bioinformatician	Developer of genomics tools	Developer of scientific tools	Senior researcher and software architect



**Computational personalised medicine competencies**	Follow the scientific method and proceed with all the steps in the process of solving a scientific problem	W	W	S	S	S	S	W	A	W	S
	Apply expertise in medical or biomedical sciences	A	W	W	A	W	S	W	A	A	N/A
	Apply expertise in formal, natural and life sciences	W	W	S	S	S	W	S	W	S	A
	Handle data from end to end following best practice	A	A	W	W	A	W	S	S	W	W
	Apply data science expertise to clinical and life sciences problems	W	A	S	W	W	W	S	W	W	N/A
	Comply with professional, ethical, legal and social standards and codes of conduct	A	A	A	A	A	S	S	W	W	W
	Design and run user-driven services and activities	N/A	N/A	N/A	N/A	N/A	N/A	W	S	W	S
**General computing competencies**	Evaluate the ability of a program running in a specific computing environment to perform a simulation (e.g. define algorithmic time and hardware resources required to solve a problem)	W	A	W	N/A	W	A	W	S	W	S
	Operate effectively within a Linux environment	W	A	W	W	W	N/A	S	W	W	S
	Write or adapt scripts and computer programs (software development) to perform simulations in compliance with good programming practice	W	N/A	W	N/A	W	N/A	W	S	W	W
	Install or deploy pre-built software on a desktop or server computer	S	A	S	A	S	A	S	S	W	S
	Acknowledge, and comply with, licensing policy	A	N/A	A	A	A	A	S	S	W	S
	Monitor application execution	W	N/A	W	W	W	N/A	S	W	W	S
	Package and distribute software	A	N/A	A	N/A	A	N/A	A	S	W	S
**Parallel computing competencies**	Use a batch job system	A	N/A	W	A	W	A	S	W	W	W
	Use computational workflow systems, understanding their potential benefits and limitations	A	N/A	W	A	W	A	S	W	W	S
	Write parallel programs	A	N/A	A	N/A	A	N/A	A	S	A	W
	Assess advantages and limitations for deploying, executing and optimising computations in a cloud/grid/HPC environment	A	N/A	W	N/A	W	N/A	W	W	W	S
	Use performance profiling to identify bottlenecks and optimise the code	A	N/A	A	N/A	A	N/A	A	S	W	S

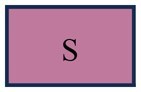
 Specialist knowledge

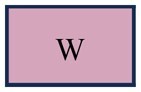
 Working knowledge

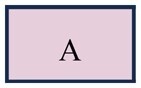
 Awareness

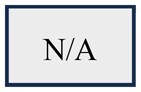
 Not applicable

In addition to the technical competencies mentioned above, professionals in computational personalised medicine require transversal competencies, such as communication, team work or management skills. Other frameworks, such as the European Competence Framework for researchers (https://research-and-innovation.ec.europa.eu/jobs-research/researchcomp-european-competence-framework-researchers_en), the ISCB competency framework (https://competency.ebi.ac.uk/framework/iscb/3.0), or the Vitae Researcher Development Framework (https://vitae.ac.uk/vitae-researcher-development-framework/), already address these competencies, so we relied on these frameworks and aligned with them instead of developing similar competencies.

PerMedCoE has used its competency framework as a tool to inform the design and development of a training programme tailored to professionals in computational personalised medicine. The framework can also be used directly by these professionals to assess and inform their professional development. To this end, we made the competency framework available on the EMBL-EBI Competency Hub (https://competency.ebi.ac.uk/), a platform that hosts competency frameworks in the life sciences and enables users to find training resources associated with specific competencies, assess themselves against these competencies, and compare career profiles. Using and sharing the competency framework is aligned with recommendation N of the bicycle principles (https://www.bikeprinciples.org/#n_career-spaning-guidance/): ‘Encourage evidence-based guidance to support career-spanning learning’ (Williams *et al.* 2023).

### 2.2 Definition of career profiles

To better understand user communities and the target audience for the training courses, PerMedCoE created a series of career profiles (often referred to as personas), which exemplify professionals within the field and include a description of the qualifications, background, and activities needed in the role. The career profiles are fictional, based on an amalgam of professionals in related roles, and developed with input from professionals in the role or similar roles. Each career profile includes: (i) a brief description of a possible career path and job activities, and (ii) information on the level of each PerMedCoE competency required to perform in the role. The current list of profiles with their competence level can be found in Table 1 and the full profiles, including the descriptions, can be found on the EMBL-EBI Competency Hub.

In each career profile, the competencies are assigned one of three levels: awareness, working knowledge, or specialist knowledge. In some cases, a competency is considered ‘not applicable’ if it is not relevant for that particular professional role. When designing training programmes and courses, the career profiles help us to define the target audience and therefore decide the content to include, and the level of competency that we are aiming to support trainees to gain or reinforce. For example, the career profile for a biomedical researcher indicates that their level of competence to evaluate the ability of a program to perform a simulation, to operate in a Linux environment or to use a batch job system is very low. Therefore, for them to be able to run simulations in HPC machines, it is necessary to acquire those skills starting at a beginner level. That was the main purpose of one of the courses in our training programme, ‘Introduction to HPC for life scientists’, which was started by BioExcel in 2018, and co-organised by PerMedCoE in 2022 and 2023.

## 3 Training needs analysis

Ideally, a training programme to build capacity in computational personalised medicine should address all the competencies included in the competency framework, as well as some essential transversal competencies, such as communication, team work or project management. To make the most impact within the resources and timeframe of the EU-funded PerMedCoE initiative, it was essential to focus the training programme on key competencies that would deliver the greatest benefits. A training needs analysis (https://permedcoe.eu/wp-content/uploads/2023/03/PerMedCoE_D4.2_Trainig-needs-analysis-and-training-plan_-v1.0_final.pdf) was performed through: structured interviews with the project partners; definition of user profiles and their relationship to the competency framework, and a gap analysis based on the mapping of competencies to existing training resources.

The conclusions from the training needs analysis were that training should be prioritised in three main areas: ‘modelling’, ‘HPC’ and ‘parallel programming’. Different types of audience require training at different levels within those areas: for example, a clinical geneticist might need to explain how modelling can inform medical decisions, while a computational biologist might require in-depth training on the use of specific modelling tools. Therefore, the PerMedCoE training programme prioritised two streams of training. The first one focused on the training needs of the consortium members (Table 2, internal training), and enabled the consortium to build capacity to deliver PerMedCoE’s objectives. The second stream was aimed at biomedical researchers (Table 2, external training), enabling them to perform modelling and simulations of cellular systems and to become users of the PerMedCoE environment. It included foundational training on HPC, PerMedCoE workflows, and user training on CellNOpt (Terfve *et al.* 2012), CARNIVAL/CORNETO (Liu *et al.* 2019, Rodriguez-Mier *et al.* 2025), MaBoSS (Stoll *et al.* 2017), COBREXA (Kratochvíl *et al.* 2022), PhysiCell (Ghaffarizadeh *et al.* 2018), and PhysiBoSS (Letort *et al.* 2019).

**Table 2:** Mapping of competencies to the live training courses delivered as part of the PerMedCoE training programme. Shaded cells indicate the competencies that each course contributes to develop.

		Internal training		External training				
		Workshop on Building blocks, Workflows and Containers design	Assessing the parallel application performance of PerMedCoE tools using the POP methodology	Introduction to HPC for life scientists	Drug studies in signalling pathways models and their integration into multiscale models	Systematising complex and combined metabolic analyses with COBREXA.jl	From transcriptomics to mechanistic models of signalling	PerMedCoE summer school: from pathway modelling tools to cell-level simulations
**Computational personalised medicine competencies**	Follow the scientific method and proceed with all the steps in the process of solving a scientific problem							
	Apply expertise in medical or biomedical sciences							
	Apply expertise in formal, natural and life sciences							
	Handle data from end to end following best practice							
	Apply data science expertise to clinical and life sciences problems							
	Comply with professional, ethical, legal and social standards and codes of conduct							
	Design and run user-driven services and activities							
**General Computing competencies**	Evaluate the ability of a program running in a specific computing environment to perform a simulation (e.g. define algorithmic time and hardware resources required to solve a problem)							
	Operate effectively within a Linux environment							
	Write or adapt scripts and computer programs (software development) to perform simulations in compliance with good programming practice							
	Install or deploy pre-built software on a desktop or server computer							
	Acknowledge, and comply with, licensing policy							
	Monitor application execution							
	Package and distribute software							
**Parallel computing competencies**	Use a batch job system							
	Use computational workflow systems, understanding their potential benefits and limitations							
	Write parallel programs							
	Assess advantages and limitations for deploying, executing and optimising computations in a cloud/grid/HPC environment							
	Use performance profiling to identify bottlenecks and optimise the code							

## 4 The training programme

**Figure 1:**
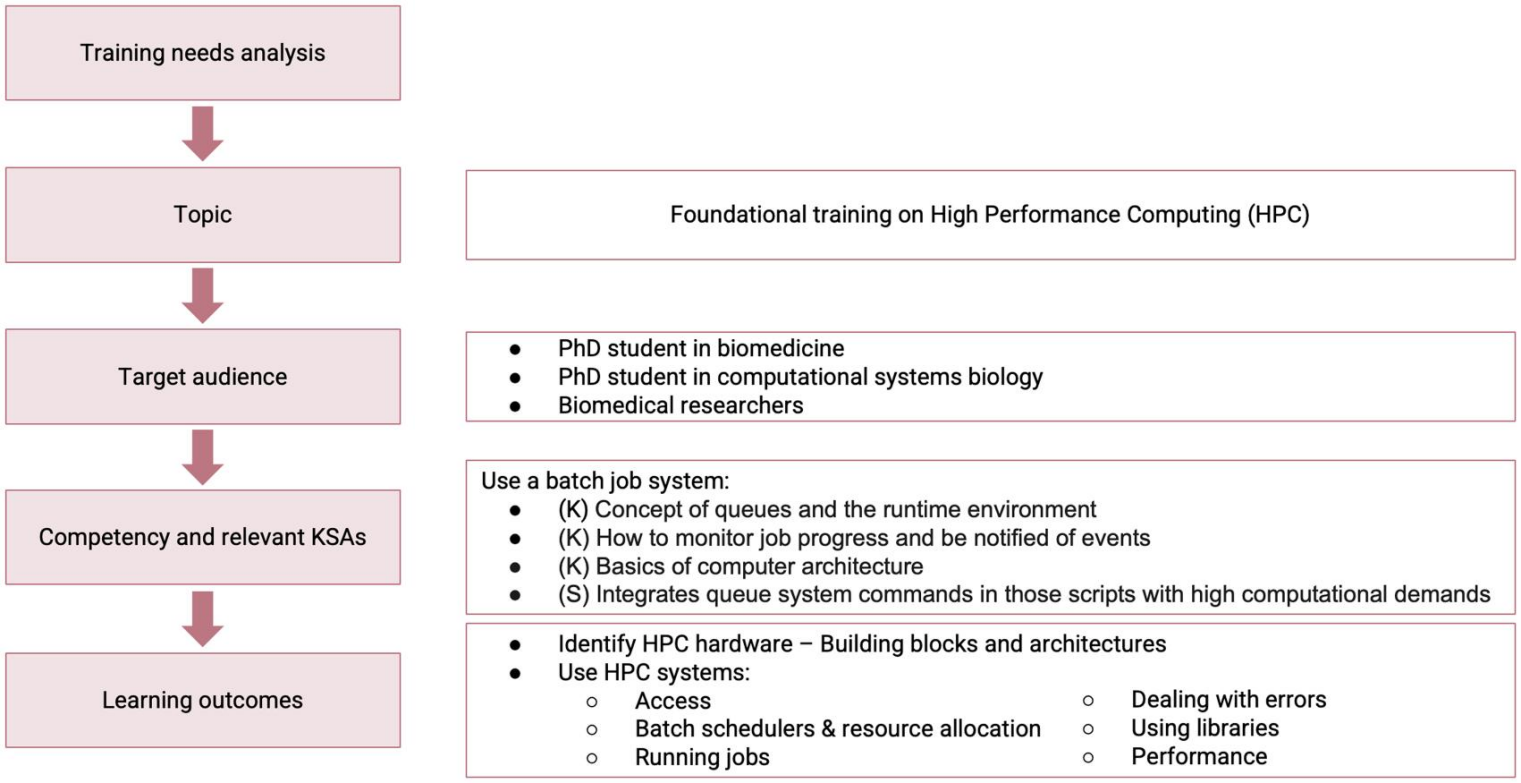
Design of a training course: illustrative example with a subset of a competency. The training needs analysis indicated a need to deliver foundational training on High Performance Computing (HPC), so we designed and delivered a course entitled ‘Introduction to HPC for Life Scientists’. The relevant target audience in the context of PerMedCoE were PhD students in biomedicine and in computational systems biology. One of competencies for them to develop in relation to HPC is ‘Use of a batch job system’. Some relevant atributes from this competency are highlighted here, as they serve as the basis to decide learning outcomes for the course: ‘Identify HPC hardware’ and ‘Use HPC systems’. The design of a course includes this process for multiple competencies and takes into account the context of the project, such as available resources or strategic priorities.

The PerMedCoE training programme focused on the priority areas identified above, ‘modelling’, ‘HPC’ and ‘parallel programming’, and aimed at developing the relevant competencies related to these topics according to the target audience for each course. The design and delivery of the programme, and the activities incorporated into the courses, were guided by best practice in training and recommendations such as the bicycle principles for short-format training (Williams *et al.* 2023) and built on the expertise from the EMBL-EBI Training Team and various schools ran during the BioExcel project (Mendonca *et al.* 2022). The information on the competency framework was incorporated in the courses following the guidelines from ISCB (Schwartz *et al.* 2012), as exemplified in Fig. 1. A dissemination strategy was designed in parallel with the training programme to reach our target audiences. Events and materials were shared through the project website (https://permedcoe.eu/), LinkedIn (https://www.linkedin.com/company/permedcoe/about/), and X (https://x.com/PerMedCoE) accounts.

**Figure 2:**
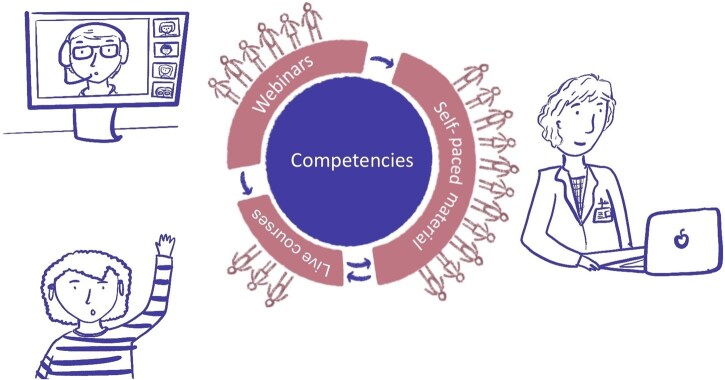
The PerMedCoE training programme. The PerMedCoE training programme is based on competencies and it includes three types of activities related to each other: the materials used for live courses can be adapted as self-paced learning material, which, in turn, can be used as pre-reading material for live courses or as the basis for some sessions; the materials for a live webinar can also be used in the other two types of activity; webinar recordings become available online after the live event. The reach achieved with these activities vary: live courses are for a limited number of people, which is larger for webinars, while materials available online have a much broader reach. Original drawings by Juanita Riveros Cuestas, EMBL-EBI.

The PerMedCoE training programme included synchronous events, including webinars, virtual and face-to-face training courses, and asynchronous, self-paced online learning material. All of these training modalities are too short to cover all the attributes of a competency, but they contribute to developing them (Table 2). The combination of formats enabled us to reach a broad audience with a diverse range of availability, preferences, and needs. Different formats can complement each other and enrich the training offer and the learning experience of the users, for example: a pre-recorded webinar can be used as preparatory material for a synchronous course, and material from a synchronous course can be published so that anyone can follow it at their own pace (Fig. 2).

### 4.1 Live events

A regular webinar series showcased relevant topics for the computational personalised medicine community and served as a good introduction to a wide range of topics: the use of specific cell-level simulation tools, applications of simulations in research, and presentation of technical developments. The webinar series contributed to raising awareness about PerMedCoE activities and facilitated our participation in the scientific conversation through the invitation of external experts.

Live training courses combined theoretical and practical sessions and included time for discussion and networking in the form of breaks, poster sessions, or social activities. Participants and trainers had the opportunity to exchange ideas about their research or the development of the tools that were presented.

The training for the consortium members included courses that developed competencies in the parallel computing domain such as ‘monitor application execution’ or ‘write parallel programs’. The target audience consisted of software developers who needed to improve their skills in parallel programming, as found in the training needs analysis. One of these courses, ‘Assessing the parallel application performance of PerMedCoE tools using the POP methodology’, was organised through a collaboration with the Performance Optimisation and Productivity Centre of Excellence in HPC (POP CoE, https://pop-coe.eu/) to deliver the content, and to provide support to PerMedCoE developers. In addition to events focused on computational skills, a Train-the-trainer course was organised to align practice, prepare experts to address trainees with a diverse set of skills, and increase the capacity within PerMedCoE to deliver the training programme.

The training needs analysis indicated that the potential users of the simulation software developed by PerMedCoE required foundational training on HPC and on the use of modelling tools. Therefore, we developed a series of short online courses, of a duration between half a day and three days, aimed at biomedical professionals external to the consortium. The focus was on developing competencies related to these topics, such as ‘use a batch job system’, ‘apply data science expertise to clinical and life sciences problems’, or ‘evaluate the ability of a program running in a specific computing environment to perform a simulation’. The material developed for these courses and the lessons learned when delivering them informed the design of a longer course, the PerMedCoE summer school (explained in more detail below), where we trained participants in the use of all the simulation tools developed by the consortium.

### 4.2 Self-paced learning materials

PerMedCoE’s live training events were complemented with a collection of online material openly available on the PerMedCoE website (https://permedcoe.eu/training_type/material/) that includes material on basic computing skills, usage of HPC, and on the tools that the consortium focused on: CellNOpt (Terfve et al. 2012), CARNIVAL (Liu *et al.* 2019, Rodriguez-Mier *et al.* 2025), and MaBoSS (Stoll *et al.* 2017) to characterise cell signalling under different formalisms, COBREXA (Kratochvíl *et al.* 2022) to quantify cell metabolism through constraint-based modeling, or PhysiCell (Ghaffarizadeh *et al.* 2018) and PhysiBoSS (Letort *et al.* 2019) to simulate multicellular systems and their environments with multiscale agent-based modelling. Interactive notebooks were used whenever possible, as they integrate code and explanations and allow learners to gain hands-on experience with the tools. Part of this material was created specifically as self-paced learning material, and some was adapted from the material used on the live courses, which multiplied our reach, and allowed us to capitalise on the large effort required to develop the live courses. Webinar recordings and slides were shared after each event, so they are available to anyone interested in learning about (or indeed teaching about) a variety of topics related to computational personalised medicine.

When creating and sharing training materials, we have been guided by the FAIR principles (Garcia *et al.* 2020) and our commitment to making science open and accessible to everyone following the UNESCO recommendations (https://www.unesco.org/en/open-science). In most cases, the materials are shared under a creative commons CC-BY 4.0 licence (https://creativecommons.org/licenses/by/4.0/), and many of the tutorials are available in GitHub repositories (https://github.com/PerMedCoE) or published via Read the Docs pages (https://permedcoe.readthedocs.io/en/latest/03_existing/existing.html). To improve the sustainability, we published some of the documentation as reproducible workflows implemented using the literate programming methodology (Pieterse *et al.* 2004). Such workflows are automatically tested for complete reproducibility upon each software release using continuous integration tools, and deployed to GitHub Pages. This approach rules out the possibility of documentation mistakes such as missing and outdated function descriptions, improving the user experience. In addition to the PerMedCoE website, training materials were added to the ELIXIR TeSS (Training eSupport System, https://tess.elixir-europe.org/), a repository for bioinformatics training material, with a detailed description and relevant metadata.

### 4.3 PerMedCoE summer school: from pathway modelling tools to cell-level simulations

**Figure 3:**
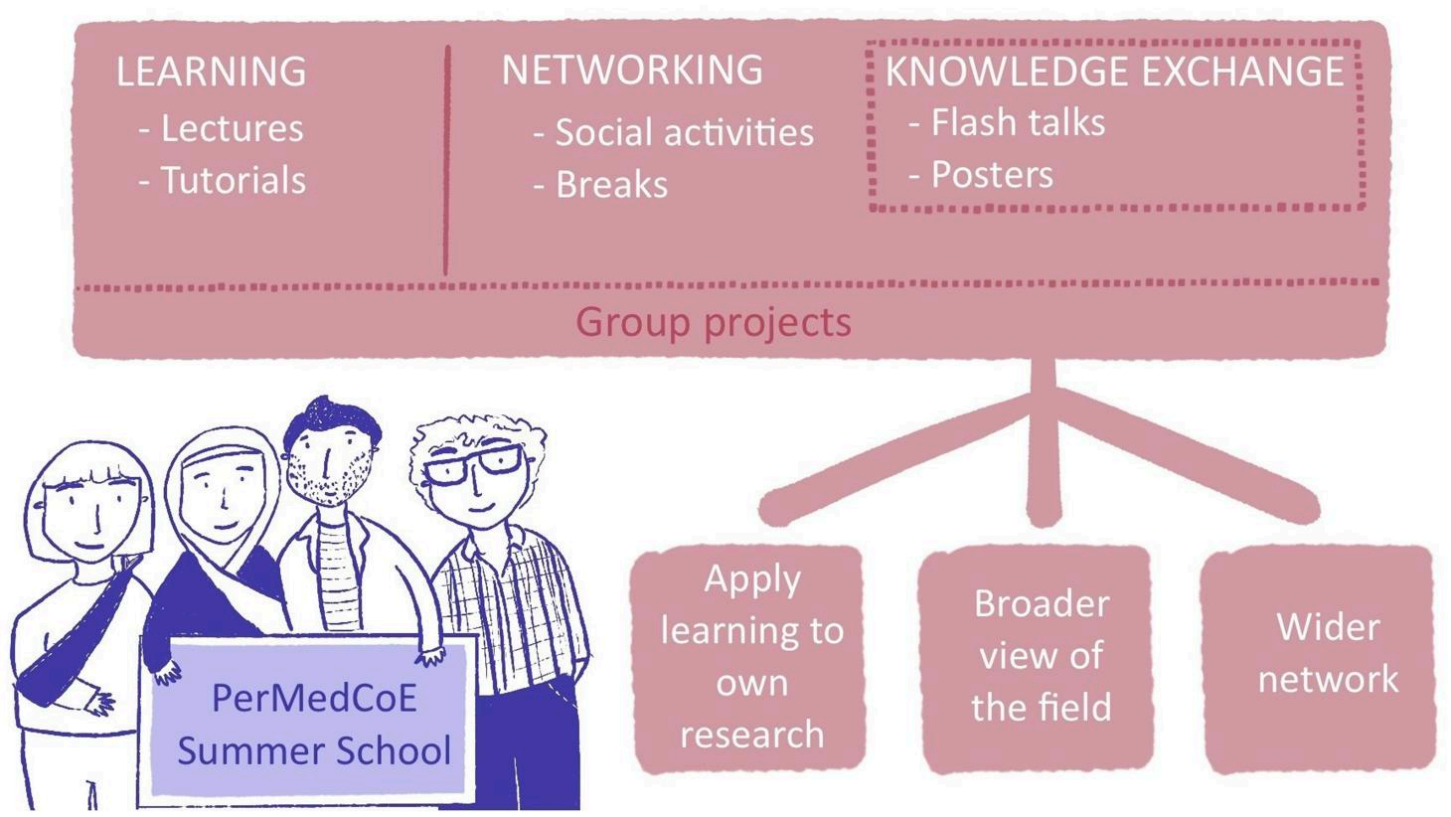
The PerMedCoE Summer School. The course included different types of activities to facilitate learning, networking, and knowledge exchange. A course like this is expected to impact participants in different ways: applying new skills to their own research, expanding their view of the research field, and providing a wider network within and beyond their field of expertise. Original drawings by Juanita Riveros Cuestas, EMBL-EBI.

This event was the flagship of the PerMedCoE training programme in terms of upskilling biomedical researchers in the areas of HPC and the use of modelling tools, leveraging on the work done throughout the project. The summer school was designed as a 5-day long residential course where all parties involved—participants, trainers, and organisers—were hosted together in the same venue. This model facilitates formal and informal interactions, contributing to the consolidation of a community around the project and the field. The programme consisted of lectures, hands-on sessions, flash talks and poster sessions, a career session, and networking activities (Fig. 3). The materials were made openly available after the course (https://www.ebi.ac.uk/training/materials/permedcoe-summer-school/).

The PerMedCoE competency framework informed the content of the lectures and hands-on tutorials, which focused on competencies such as ‘Apply data science expertise to clinical and life sciences problems’, ‘Evaluate the ability of a program running in a specific computing environment to perform a simulation’ or ‘Use computational workflow systems, understanding their potential benefits and limitations’. The delivery of shorter courses beforehand allowed the trainers to design the sessions for the Summer School based on their previous experience and the participants’ feedback, making the organisation of this major training event manageable in the context of a time-limited project, and aligned with the progress of the tools and software development within the project.

In addition to sessions about specific tools and methods, the course included time for participants to apply their learnings to a specific project inspired by real research scenarios. This allowed them to practice by themselves in small groups and assess their knowledge and skills, under the direct guidance of the tool experts. Both the project guidance and the materials from each session contained links to further reading and the tools documentation to facilitate self-directed learning once participants were back to their research and wanted to apply the tools to their own research questions. These activities, therefore, aligned with the following recommendations of the bicycle principles for short-format training (Williams *et al.* 2023): ‘Develop an implementation strategy for Catalytic Learning’, and ‘Support integration of diagnostic assessment into short-format training’ (recommendations L and M).

All participants prepared a poster about their research, which was on display during the entire week of the course. This, together with the two poster sessions included in the programme and the group projects, facilitated interaction, scientific discussion, and brainstorming with other participants, including the trainers. Networking was enhanced by flash talks delivered by both participants and trainers following a ‘2 slides in 2 minutes’ format, where both the individual and their research were presented.

The impact that the summer school had in the following months reflects the consolidation of the community. Several members connected with others through social media, live events (e.g. ISMB 2023), or through the PerMedCoE webinar delivered by the three poster winners of the Summer School (https://permedcoe.eu/training/webinar-poster-winners-permedcoe-summer-school-2023/). Furthermore, the impact survey results show how attendance to the course improved their research, as explained in detail in the following section.

## 5 Impact of the training programme

The PerMedCoE training programme has empowered life scientists to run cell-level simulations in HPC environments and apply advanced modelling tools to characterise cell signalling and metabolism. It has reached over 2000 participants through live activities, almost 8000 viewers have accessed the recorded videos, and the training materials webpage got more than 1300 views and more than 4200 event counts as per the end of June 2025 (data collected via Google Analytics 4). Through the training programme, PerMedCoE has positioned itself as a training reference in the field of computational personalised medicine.

The impact of the training programme is tangible when considering that some researchers decided to attend other training activities after their first contact with the PerMedCoE training programme. Some participants of the training programme have reported that they gained knowledge and skills that helped them in their work and how they approach their research, e.g. improving their computational thinking, characterising signalling pathways in their own research, learning how to integrate different levels of biological systems. In addition, thanks to the networking platforms put in place for the courses, attendees established connections with participants and trainers beyond the frame of the PerMedCoE events, which can be valuable in their research careers.

These conclusions are supported by the data collected through the feedback and impact surveys. The quality of the courses was assessed through a survey at the end of each course. The course feedback surveys showed that, on average, 86% of the respondents rated our courses as very good (4 out of 5) or excellent (5 out of 5) in a ranking scale of 5, and 96% of them would recommend the course.

The course feedback survey provides information about how the course was perceived by participants, but it cannot inform on the longer term impact on the participant’s work and career. Therefore, long-term impact surveys were sent between 6 months and 1 year after each course to collect information on how participants applied the learning to their research, interactions, or collaborations with others at the workshop and whether they have taught others. We received 19 complete (11% of all participants who received the survey) and 16 incomplete surveys (9%). These completion rates are in line with values from other courses at EMBL-EBI and ELIXIR. 74% of the respondents continued using the methods and resources they were trained on, and 68% cascaded the knowledge and skills gained to others. More than 40% of the respondents interacted with the course peers or trainers after the event and found the course useful to establish collaborations or to connect with communities working in the field. Finally, 95% of the respondents would recommend or had already recommended the course, which serves as an indication of the high quality of the training activities organised by PerMedCoE.

## 6 Conclusions

New methods and technical developments are constantly evolving in the life sciences and therefore it is essential for researchers to keep abreast of them. Short training interventions can contribute to this upskilling, but for them to be effective and meaningful, it is necessary to take into account the characteristics and needs of the target audience, and to maximise opportunities for participants to reinforce their learning in the context of their own research. Therefore, the identification of, and engagement with, relevant stakeholders is essential.

PerMedCoE adopted a competency-based approach to develop its training programme, which provided a structured framework to develop training courses and materials that could be used on their own or in combination to upskill life sciences researchers in the use of modelling methods and tools. In addition, the approach includes the definition of competencies and career profiles, which can be used by professionals and employers in the community to guide professional development and hiring strategies.

In the context of time-limited projects that develop new tools for cutting-edge science, it is often challenging to develop impactful training programmes with a wide reach. The amount of short training interventions that can be delivered is limited and cannot cover all the competencies included in the framework or cover them at depth, but developing a competency framework helps to structure and prioritise the activities in the learning programme to maximise impact, and provides a foundation upon which future training can be developed. Researchers who participate in the programme understand which competencies they are developing and which ones they need to develop further, which can be pursued after the end of the funded project by other means. Furthermore, the alignment and collaboration with other HPC projects, like BioExcel, POP CoE, and CompBioMed contributed to an efficient use of resources to organise training activities.

The success of the PerMedCoE training programme was key in positioning PerMedCoE as a reference in the field of cell-level simulations, and enhanced the competence level of the PerMedCoE toolkit users. Following this approach helped to increase capacity within the consortium and to establish a community around a project, by planning regular and diverse activities that build and maintain a momentum in the field and, therefore, keep the users and other stakeholders engaged. This was facilitated by a good dissemination and communication strategy to connect with the community and the variety of platforms used to share our materials: PerMedCoE website (https://permedcoe.eu/), GitHub repository (https://github.com/PerMedCoE) and YouTube channel (https://www.youtube.com/channel/UClZV7luI2oU-TV-jKtEPzKw), TeSS (https://tess.elixir-europe.org/), EeLP (https://elixir.mf.uni-lj.si/), and EMBL-EBI training website (https://www.ebi.ac.uk/training/).

The competency-based approach used by PerMedCoE supported the project to define stakeholders’ competency requirements, build a programme based on PerMedCoE’s unique combination of advanced biomedical and computational expertise, and support community development, both within and beyond the confines of the project. Furthermore, the outputs from the PerMedCoE training programme—the competency framework, the self-paced online courses, and training materials—are openly available for reuse and further development by others, contributing to the sustainability of the project’s outputs. We believe that our experience of developing the PerMedCoE training programme could readily be applied in other contexts of continuous innovation where the workforce needs regular training to stay up to date with new tools and developments.

## Supplementary Material

vbag070_Supplementary_Data

## Data Availability

The competency framework is reproduced in full in this paper as supplementary material and available on the Competency Hub at https://www.ebi.ac.uk/training/. Information on the training resources is available in the PerMedCoE website (https://permedcoe.eu/) and EMBL- EBI Training website (https://www.ebi.ac.uk/training/).
