# Supplementary material for: Leveraging training expertise to build capacity in computational personalised medicine
Source: Bioinform Adv. 2026 Apr 25;6(1):vbag070. doi: 10.1093/bioadv/vbag070 (PMC13110006; doi:10.1093/bioadv/vbag070)
Supplement: vbag070_Supplementary_Data [file vbag070_supplementary_data.zip › 30-Mar-2026_102724_Supplementary_table_competencies_PerMedCoE.docx]

**Supplementary Table 1. PerMedCoE competency profile Version 2.0**

|  | **Computational personalised medicine competencies (CPM)** | | | |
| --- | --- | --- | --- | --- |
| **Code** | **Competencies** | **Knowledge** | **Skills** | **Attitudes** |
| CPM1 | **Follow the scientific method and proceed with all the steps in the process of solving a scientific problem** | - Principles of the scientific method, including hypothesis generation  - Experimental design  - Relevant data sources, types and standards  - Analysis methods and tools available appropriate to the research question  - Relevance of replicates and positive and negative controls  - Experimental validation | - Locates and critically assesses the accuracy and reliability of relevant published works and data  - Designs experiments in a critical way to answer research questions  - Applies methods and technologies appropriate to the research question  - Performs experiments and analysis according to best practice in the field  - Critically assesses the results obtained throughout the experimental process  - Draws conclusions based on the data and the methods employed | - Takes into consideration prior work at all steps of the experimental process  - Creatively addresses research gaps and needs  - Critically evaluates new technologies and discoveries  - Uses relevant benchmarking environments supported by the research community  - Reconsiders hypotheses on the basis of unexpected results  - Documents negative results and shares them with the community |
| CPM2 | **Apply expertise in medical or biomedical sciences** | - Main aspects of biomedical and clinical problems to a detailed level  - Extensive scientific and/or clinical knowledge  - Principles of translational medicine, medical genomics, and drug discovery as appropriate  - Applications and limitations of bioinformatics in a medical context  - Gaps between research and medical applications | - Has an interdisciplinary view  - Applies relevant methods to clinical questions  - Interprets results appropriately in the clinical context and sets them in the context of previous scientific and clinical knowledge | - Takes a comprehensive approach to clinical problems  - Integrates ideas from the broader clinical community  - Embraces collaboration with other clinicians and researchers and communicates effectively with them  - Demonstrates critical and creative thinking and encourages it in others  - Appreciates the impact of results in patients and their families  - Translates the real-world needs to actionable research and development topics |
| CPM3 | **Apply expertise in formal, natural and life sciences** | - Main aspects of biological problems to a detailed level  - Scientific knowledge in at least one of the following areas: biology, chemistry, physics, mathematics  - Deep domain knowledge in specific topic  - Core principles of systems biology  - Current challenges in their scientific community  - Applications and limitations of relevant methods and technologies  - How different methods and technologies can validate and/or complement each other | - Has an interdisciplinary view  - Asks relevant, hypothesis driven, well-defined biological questions  - Plans and performs experiments responsibly  - Interprets experimental results appropriately and sets them in the context of broader knowledge | - Takes a comprehensive approach to scientific problems  - Integrates ideas from the broader scientific community  - Embraces collaboration at both intra- and interdisciplinary levels  - Considers the broader impact of research in the field  - Demonstrates critical and creative thinking and encourages it in others |
| CPM4 | **Handle data from end to end following best practice** | - Different types of data and databases used in their field of expertise  - Procedures for data stewardship according to relevant ethical and legal standards  - The FAIR principles for scientific data management  - The data management policy of the institution/compute resource  - The role of governance, curation and information architecture in data management  - Common document identification, tracking and control procedures  - Data representation including file formats, ontologies and other controlled vocabularies  - Data storage and format requirements of downstream techniques to integrate, interpret, analyse and visualise biological data sets | - Locates relevant data from different sources and assesses its quality (e.g. methods, data, results)  - Drafts and files an appropriate data management plan  - Handles data according to standard procedures: moves data safely, documents every step and has backup and version control systems in place  - Stores and analyses data in accordance with ethical, legal and commercial standards, including checking who has access  - Curates biological data using suitable metadata, ontologies and/or controlled vocabularies  - Submits data to appropriate public data repositories as required, being aware of format requirements and of ethical and legal considerations | - Follows and promotes the FAIR principles in the data management cycle  - Actively promotes and uses existing standards, ontologies and metadata annotations  - Advertises the existence of good quality datasets to others  - Contributes to public datasets when appropriate  - Acts with awareness of the wider context in which scientific research operates, recognising the implications for professional practice |
| CPM5 | **Apply data science expertise to clinical and life sciences problems** | - General data science approaches to life sciences problems, including machine learning and artificial intelligence when appropriate  - Statistical and mathematical modelling methods, and key scientific and statistical analysis software packages  - Appropriate statistics in the context of -omics or systems biology data analysis  - Experimental design to ensure the statistical validity of high-throughput experiments | - Determines the best methods for data analysis, including the selection of statistical tests, considering the research question and limitations of the experimental design  - Designs appropriately powered experiments to answer the research question  - Applies statistical methodologies appropriate to the analysis of data in the context of the life sciences | - Approaches problems with a systems-based, data-driven approach to scientific discovery  - Appreciates the importance of statistics in experimental design, data analysis and interpretation  - Recognises own limitations and consults experts when required  - Is conscious of the risks of overfitting  - Appreciates the importance of sharing statistical methods applied in a project, the rationale, limitations and results |
| CPM6 | **Comply with professional, ethical, legal and social standards and codes of conduct** | - Potential implications of bioinformatics work for the rights of individuals, groups and populations  - Requirements for responsible, legal or ethical access and use of life sciences data including relevant data protection regulations, and standards for access and benefit sharing  - Genome and healthcare data security and privacy issues including the possibility to re-identify individuals based on their data  - Codes of conduct and standards of publication ethics relevant for the profession  - Fundamental principles of intellectual property rights and associated policies as they apply for data(bases), publications, software and research results  - Licensing practices for (open source) software and (open) data | - Obtains data sets from private and/or public resources considering any legal, privacy or ethical aspects of use  - Recognises potential ethical issues of bioinformatic and/or genomic studies, datasets, or conclusions drawn from their analysis  - Maintains requisite data privacy, security, and IP rights when storing, manipulating or submitting data  - Practices objectivity in their professional activities through recognition, acknowledgement and mitigation of intentional and unintentional biases | - Interprets relevant standards and codes of conduct judiciously and seeks expert advice when needed  - Treats all fellow professionals, regardless of grade, with equal respect and refrains from all discrimination  - Respects confidentiality and privacy, and ensures that other team members and collaborators respect it  - Confronts potentially unethical behaviour or behaviour at odds with the organisation’s values  - Fosters fair participation of all people and strives to enhance the quality of life of those affected by their work |
| CPM7 | **Design and run user-driven services and activities** | - Types of users, their interests and motivations  - Activities and workflows employed by the users  - User experience design  - Characteristics and behaviour of the (potential) user communities | - Collects user needs and requirements  - Differentiates wants from needs  - Manages expectations of users and user communities  - Designs and organises services and activities considering the relevant user needs  - Keeps the users informed about the novel implementations based on their input | - Has a user-oriented mindset  - Empathises with (potential) users and user communities  - Seeks out and acts on user feedback to improve the user experience and increase satisfaction |

|  | **General Computing competencies (C)** | | | |
| --- | --- | --- | --- | --- |
| **Code** | **Competencies** | **Knowledge** | **Skills** | **Attitudes** |
| C1 | **Evaluate the ability of a program running in a specific computing environment to perform a simulation (e.g. define algorithmic time and hardware resources required to solve a problem)** | - Capabilities and limitations of computer-based systems, processes, components and programs  - Software requirements of the program  - Optimal hardware to run the program  - Where to find information about the level of trust of the system and its suitability for work with sensitive data  - How to describe algorithmic performance and complexity (e.g. Big O notation) | - Evaluates applicability, optimisation and scope of different tools before choosing  - Is able to benchmark a program to get an estimate of the required computing time  - Identifies bottleneck in the program (network, CPU, RAM, etc)  - Assesses when to move data, and when to run code elsewhere | - Keeps up to date with emerging techniques and applications  - Actively searches for available sources of support (training material, forum, helpdesk, etc.)  - Prototypes/implements the design solution and verifies performance against specification  - Checks output correctness according to software documentation  - Identifies new technological opportunities |
| C2 | **Operate effectively within a Linux environment** | - How to efficiently navigate their way around the Operating System (OS), including the file system  - Where to find the location of important configuration files & applications  - Most common CLI tools / programs (grep, find, du, etc.) | - Creates and manages files and directories in a system  - Is able to use Unix/Linux features like pipes & redirection  - Changes access permissions when required  - Searches effectively for files and content  - Reads and edits files without a GUI  - Writes scripts to automate and/or facilitate actions when appropriate | - Searches proactively for support on the web  - Creates backup files  - Uses access permissions appropriately  - Is up to date with new bug fixes and security patches from periodic Linux updates |
| C3 | **Write or adapt scripts and computer programs (software development) to perform simulations in compliance with good programming practice** | - Common programming concepts like loops and function calls  - The pros and cons of different scripting and programming languages  - Existing tools and libraries to reuse  - Where to find examples or written guidelines regarding best practice in their field  - The importance of writing an optimised code | - Reads, adapts and debugs existing programs or scripts  - Judges when a task should be automated or scripted  - Compiles code when appropriate  - Designs and structures efficient and portable scripts and programs  - Writes and runs appropriate tests  - Uses a compiled / high-performance language (e.g. C++, Julia) in contrast to scripting/interpreted language (Python/Bash) when needed | - Understands the need for best practice  - Conforms, and inspires others, to best practices for writing reusable code  - Uses revision control, including code review  - Uses an editor with support for programming  - Uses appropriate scripting or programming language |
| C4 | **Install or deploy pre-built software on a desktop or server computer** | - Existing repositories and revision control systems (e.g. Git, SVN, mercurial-versioning)  - The stages of software release cycles  - How to install software and dependencies  - The dependencies of the software  - The difference between kinds of binaries & source  - The different types of account (e.g. user, admin) and when they should be used  - Command line use | - Selects appropriately packaged code  - Uses package managers  - Is able to revert a system to a known state  - Debugs (Mission Control, VisualVM) and tests software  - Accesses servers remotely | - Consults the manual  - Checks licensing before installing or running software  - Appreciates the impact of installing or running software on other users of the machine  - Debugs and tests non-commercial software that is built collaboratively on a volunteer basis |
| C5 | **Acknowledge, and comply with, licensing policy** | - Types of licensing and differences between them  - Significance and potential ambiguity of licensing depending on where you work (e.g. academia/industry, funding)  - The difference between open and closed licenses | - Chooses appropriate license for their own software  - Identifies tricky or unclear licenses and seeks expert help  - Keeps track of licensing terms of dependencies | - Always checks the license information for software  - Questions ambiguity in license information |
| C6 | **Monitor application execution** | - The relevant metrics to monitor ​​(e.g. queue monitoring, storage used)  - Which metrics require special permissions to monitor  - A variety of monitoring tools and their usage model (e.g. has to be started with the application, only targets a single process) | - Recognises bottlenecks in the program (Network, CPU, RAM, etc.)  - Identifies the current state of a process  - Checks the resource utilisation (e.g., memory, CPU) for a process | - Uses resources efficiently  - Actively considers impact on the platform and on other users |
| C7 | **Package and distribute software** | - The different uses for source and binary distributions  - Several packaging technologies (e.g. RPM, wheels, conda-packages) and distribution channels (e.g. distribute from your own GitHub, create a conda channel, submit code to some upstream repository) and how to package software for them  - The components required to set up a pipeline for automatic software packaging  - Main points when writing a tool documentation or tutorial  - When to package as a library vs. stand-alone executable  - Container technologies (e.g. Docker, Singularity) | - Submits a package to some upstream repository  - Automates their software packaging  - Designs a build pipeline  - Writes clear documentation providing installation instructions and adequate examples to illustrate the use of their tool | - Writes a how-to-do guide from the user perspective  - Writes software with packaging requirements in mind and focus on reusability |

|  | **Parallel computing competencies (PC)** | | | |
| --- | --- | --- | --- | --- |
| **Code** | **Competencies** | **Knowledge** | **Skills** | **Attitudes** |
| PC1 | **Use a batch job system** | - Concept of queues and the runtime environment  - Relevant CLI parameters of the available queue system  - Versions, incompatibilities and interdependencies of installed software  - How to monitor job progress and be notified of events  - Basics of computer architecture | - Integrates queue system commands in those scripts with high computational demands  - Can estimate what resources are required per job and verify or adjust the requirements after a completed job  - Avoids usage which taxes the batch job system too much  - Can estimate which software is responsible for the observed issues (e.g. is it my software or theirs which is causing the issue?)  - Identifies storage requirements and utilises reusability of the intermediate data objects/analysis step results | - Seeks optimal utilisation of the available HPC resources  - Monitors resource consumption, checks allocation on smaller tasks before submitting a big one |
| PC2 | **Use computational workflow systems, understanding their potential benefits and limitations** | - The existence and functionality of workflow systems  - Advantages and disadvantages of different workflow systems  - When (and when not) to implement workflow tools  - How workflow systems affect system usability and stability | - Evaluates and selects an appropriate workflow system  - Installs workflow managers  - Is able to run a workflow  - Writes and modifies a workflow  - Identifies the best resources to run the workflow (e.g. CPU, GPU, hybrid) | - Seeks out and makes use of appropriate existing workflows and workflow components  - Keeps in mind portability and reusability when developing a workflow |
| PC3 | **Write parallel programs** | - Common parallel programming paradigms and technologies (e.g. shared memory vs distributed memory, task-based parallelism, OpenMP, MPI, PGAS)  - Common bottlenecks in parallel programming (e.g. false sharing, static scheduling)  - Different system architectures and how they impact parallel programming | - Recognises (independent) units of work and potential parallelism and any dependencies  - Uses appropriate tools to debug, profile, refactor parallel code  - Selects the appropriate level of parallelism abstraction considering available frameworks and native language constructs  - Writes the code avoiding the most typical bottlenecks | - Makes use of automated benchmarking and regression test to monitor the performance on different types of architecture  - Actively uses profiling tools to detect parallel bottlenecks |
| PC4 | **Assess advantages and limitations for deploying, executing and optimising computations in a cloud/grid/HPC environment** | - Concepts related to virtualisation  - Use cases that are limited by computational infrastructure and would benefit from HPC and/or HTC technologies  - Upsides and downsides of the different environments | - When needed, undertakes a cost-benefit analysis to understand whether cloud resources are the best value  - Accurately judges added value of HPC/HTC technologies for different scenarios and user groups  - Is able to package software/data and deploy to the cloud  - Selects an appropriate grid or cloud provider  - Can build and provision a Virtual Machine (VM) | - Seeks out and makes use of appropriate existing virtual machines that are suitable for the scientific problem to be solved  - Evaluates whether a specific use case can benefit from the use of HPC or HTC |
| PC5 | **Use performance profiling to identify bottlenecks and optimise the code** | - Tools to help measure performance  - How to recognise the most common types of performance bottlenecks (memory, CPU or IO bound)  - How to interpret the results of performance profiling tools to modify the original code to diminish the impact of identified bottlenecks | - Is able to profile a code  - Refactors code in order to remove bottlenecks  - Is able to decide when to optimise an existing algorithm or when to change the algorithm itself | - Runs programs using appropriate resources (number of processors, etc.)  - Actively collects and analyses logging & performance information  - Foresees possible bottlenecks during the development |
